# Analysis of PD-1/PD-L1 variations in lung cancer and association with immunotherapeutic efficacy and prognosis: A nonrandomized controlled trial

**DOI:** 10.1016/j.clinsp.2024.100395

**Published:** 2024-06-08

**Authors:** Jun Ma, JianRui Song, LiNa Han, Wen Zhou, LiFeng Meng, JianHui Li, XiaoMing Bai

**Affiliations:** a Department of Thoracic Surgery, Shanxi Provincial People's Hospital, Shuangta Temple Street, Taiyuan City, Shanxi Province, China; b Wisdom Lake Academy of Pharmacy, Xi'an Jiaotong-Liverpool University, Suzhou City, Jiangsu Province, China; c Office of Health Emergency, Shanxi Provincial People's Hospital, Fifth Clinical Medical College, Shanxi Medical University, Taiyuan City, Shanxi Province, China

**Keywords:** Lung cancer, Programmed death receptor-1 (PD-1), Programmed death ligand-1 (PD-l1), Efficacy, Prognosis, Diagnosis

## Abstract

**Introduction:**

This study aims to explore Programmed Death Receptor-1 (PD-1) and Programmed Death Ligand-1 (PD-L1) variations in Lung Cancer (LC) tissues and Peripheral Blood (PPB) and their association with immunotherapy efficacy and prognosis.

**Method:**

72 patients with LC were included in the LC group and 39 patients with concurrent benign lung disease were included in the benign group. PD-1/PDL-1 was compared in PPB and lung tissue. All LC patients were treated with immunotherapy. The relationship between PD-1/PDL-1 in LC tissue and PPB and immunotherapy efficacy was analyzed. Patients were divided into death and survival groups, and PD-1/PDL-1 in tumor tissues and PPB were compared.

**Results:**

The authors found that PD-1 and PDL-1 positive expression in lung tissue and PPB in LC patients was elevated. Combined detection of PD-1 and PDL-1 was effective in diagnosing LC and evaluating the prognosis of LC patients. PD-1 and PDL-1 positive expression was reduced after disease remission while elevated in dead patients. The 3-year survival rate of patients with PD-1 positive expression was 45.45 % (25/55), which was lower (82.35 %, 14/17) than those with PD-1 negative expression. The 3-year survival rate of patients with positive and negative expression of PDL-1 was 48.78 % (20/41) and 61.29 % (19/31), respectively.

**Discussion:**

The present results demonstrated that PD-1 and PDL-1 are abnormal in cancer tissue and PPB of LC patients. The combined detection of PD-1 and PDL-1 has diagnostic value for LC and evaluation value for the efficacy and prognosis of immunotherapy.

## Highlights

• PD-1/PDL-1′s comparison in cancer tissues and PPB of the remission with the non-remission.
• Analysis of PD-1/PDL-1′s predictive value in cancer tissue and PPB on immunotherapy's efficacy.
• Comparison of patients’ 3-year survival rate with PD-1/PDL-1 in diverse cancer tissues.

## Introduction

Lung Cancer (LC), the malignant tumor with the most elevated morbidity and mortality in China, is severely invasive and numerous patients are already in the advanced stage at the time of diagnosis.^1^^,^^2^ Surgery, chemotherapy and radiotherapy, the crucial strategies for LC's cure, are available to imperatively alleviate patients’ clinical symptoms and ameliorate their quality of life, while patients’ prognosis is still unpleasing.^3^

Research has manifested that immunotherapy is available to distinctly prolong patients’ survival period.^4^ Immunotherapy strengthens immune cell function via immunosuppressants to block the co-suppression pathway, ultimately enhancing tumor cells’ killing ability.^5^ Recently, immunotherapy with Programmed Death Receptor-1 (PD-1) and Programmed Death Ligand-1 (PD-L1) as checkpoints have been broadly adopted in LC's clinical treatment. PD-1, immunoglobulin superfamily type I transmembrane glycoprotein composed of 288 amino acids, is crucially expressed on activated T-cells.^6^ PD-L1 appertains to PD-1′s ligands. Relevant reports have elucidated that variations in PD-1 and PD-L1 lead to aberrant PD-1/PD-L repressive pathways, resulting in aberrant immune function illnesses in the body. Research has illuminated aberrant PD-L1 is involved in multiple tumors’ immune escape mechanism and is nearly associated with tumors’ occurrence and advancement.^7^ PCD-1 is also associated with increased proliferation of Treg cells and enhanced immunosuppressive function.^8^ Nevertheless, few reports clearly elaborated on its association with immunotherapy's efficacy in LC patients. Consequently, this research was to explore the variations in PD-1 and PD-L1 in LC tissues and Peripheral Blood (PPB) and their association with immunotherapy's efficacy and prognosis, offering reference for the clinical cure of the illness.

## Materials and methods

### *Study design and clinical data*

#### Study design

This trial was designed as a prospective, parallel-group, nonrandomized study and followed the criteria of the CONSORT statement.

#### Clinical data

From January 2014 to April 2018, the involvement of 72 LC patients was as the LC, and the inclusion of 39 patients with benign lung diseases in the identical period was as the benign. Inclusion criteria: 1) Meet the diagnostic criteria for LC in the relevant guidelines; 2) Patients treated with PD-1/PDL-1 antibodies; 3) Patients with complete clinical data. Exclusion criteria: 1) Severe aberrant heart, liver and kidney function; 2) Patients with immune diseases like combined systemic lupus erythematosus and rheumatoid; 3) Patients with severe complications during or after surgery; 4) Patients with severe metabolic illnesses like combined hypothyroidism; 5) Patients who underwent immunotherapy prior to enrollment; 6) Patients with PD-1/PDL-1 antibodies to cure contraindications; 7) Patients with aberrant chest X-Ray or electrocardiogram.

This research was approved by Shanxi Provincial People's Hospital (Approval number: 201311S51) Institutional Review Committee and Ethics Committee. All participants received and signed informed consent.

## *Methods*

### PD-1/PDL-1′s detection in lung tissue

Taking of postoperative tumor tissue's paraffin specimen with 3 μm slice, and baking was in the oven; Dewaxing was with xylene, complete submergence of the slices was in ethylene diamine tetraacetic acid alkaline repair solution, and recovery of the antigen was implemented; Dropwise addition of non-immune goat blocking antigen was to sections, and introduction was conducted; Dropwise addition of sections was with 1 antibodies (rabbit anti-human PD-L1 monoclonal antibodies [Abcam Technology Co., Ltd., ab213524] and mouse anti-human PD-1 monoclonal antibodies [Beijing Zhongshan Jinqiao Biotechnology Co., Ltd., Beijing, China, UMAB199]); After incubating, addition of each section was into the secondary antibodies (Immunoglobulin G antibodies-hypothalamic radial glial polymer); Color development was with Diaminobenzidine solution, and determination of color development was with distilled water, Counterstaining was with hematoxylin counterstain, dehydration was with conventional alcohol gradient, fixation was with xylene, and baking was in oven. Adoption of neutral gum mount with known positive was as a control, and exertion of polybutylacrylate instead of the primary antibodies was a negative control. The location of PD-1 was in the cytoplasm with brown-yellow to tan particles appearing as positive color; PD-L1’s location was in tumor cells and interstitial immune cell membrane/intima system with brown-yellow to tan particles appearing as positive color. Table 1 for details.

**Table 1:** Comparison of general data between the two.

Classification	The LC (*n* = 72)	The benign (*n* = 39)	*χ^2^/t*	p
Gender (cases)				
Male	51	23	1.601	0.206
Female	21	16		
Age (years)	58.95 ± 6.24	57.73 ± 6.75	0.955	0.341
Course of disease (months)	15.29 ± 2.12	14.85 ± 2.36	1.003	0.318
BMI (kg/m^2^)	21.09 ± 1.87	21.67 ± 1.75	1.595	0.114
Smoking history (cases)	31	13	0.999	0.317
Pulmonary diseases’ history (cases)	62	29	2.365	0.124
Tumor's family history (cases)	9	3	0.606	0.436
Lesion site (cases)				
Left	29	17	0.114	0.735
Right	43	22		
Educational background (cases)				
High school and above	42	21	0.208	0.649
Junior high school and below	30	18		
Monthly income (cases)				
5000 yuan or more	39	26	1.629	0.202
Less than 5000 yuan	33	13		

### Examination of PD-1/PDL-1 in PPB

Collection of 5 mL fasting venous blood was from the patients; After anticoagulation treatment, the addition of d-Hanks solution with twice the volume and placing of Ficoll lymphocyte separation solution were implemented. White blood cells’ separation was conducted, extraction of total RNA was via RNA extraction kit, and test of RNA purity was via spectrophotometry (OD_260_/OD_280_ ≥ 1.8 represented superior purity). After taking 10 μL amplified product, performance of plate electrophoresis was on 2 % agarose gel covering ethidium bromide. After setting up the negative control, reverse transcription of the negative control was not implemented, observation of the positive was under an ultraviolet projection instrument, and the red-orange fluorescence band at the identical level as the positive control was identified as positive.

### Immunotherapy

All patients’ cures were with PD-1/PDL-1 antibodies (Pembroliaumab, Merck, USA) at a dose of 2 mg/kg in a three-week course with intravenous infusion. Curative efficacy assessment was in the light of Response Evaluation Criteria in Solid Tumors 1.1, and the patients’ division was into complete remission, partial remission, and stable disease progression. The patients’ division was into the remission (complete remission and partial remission) and the non-remission (stable disease and progression) on the grounds of efficacy.

### Observation indexes

(1) PD-1/PDL-1′s comparison in lung tissue and PPB in the LC and the benign was to analyze the combined detection's diagnostic value for LC. (2) PD-1/PDL-1′s comparison in LC tissues and PPB in the remission and the non-remission was to analyze the association of PD-1/PDL-1 with immunotherapy's efficacy. (3) Patients’ division was into the death and the survival in the light of 3-year survival after therapy. Two-group comparison of PD-1/PDL-1 in cancer tissues and PPB was to analyze predictive value with survival rate's relevance for patients’ prognostic.

### Statistical processing

Data processing was via applying SPSS22.0 software. Manifestation of enumeration data was in %, and comparison of the difference between groups was via exerting χ^2^ test. Through normal test, the representation of measurement data was in (means ± standard deviation), and the comparison of the difference between groups was via adopting *t*-test. Analysis of PD-1/PDL-1′s diagnostic value in lung tissue and PPB for LC was via adopting the Receiver Operating Characteristic (ROC) curve. The survival curve's rendering was with GraphPad Prism 5 software, and the test of the involved survival rate between the two was via adopting log-rank χ^2^; *p* < 0.05 was accepted as indicative of dramatical differences.

## Results

### *PD-1/PDL-1′s comparison in lung tissue and PPB in the LC and the benign*

PD-1 and PDL-1′s positive rate in lung tissue and PPB in the LC was elevated vs. the benign (*p* < 0.05), as presented in Table 2.

**Table 2:** Comparison of PD-1/PDL-1 in lung tissue and PPB of the LC and the benign (cases,%).

Groups	n	Cancer tissue PD-1	Cancer tissue PDL-1	PPB PD-1	PPB PDL-1
The LC	72	55 (76.39)	41 (56.94)	34 (47.22)	24 (33.33)
The benign	39	8 (20.51)	6 (15.38)	41 (0.26)	4 (10.26)
*χ^2^*		32.180	17.897	15.354	7.142
p		< 0.001	< 0.001	< 0.001	0.008

### *Analysis of PD-1/PDL-1′s diagnostic value in lung tissue and PPB for LC*

**Fig. 1:**
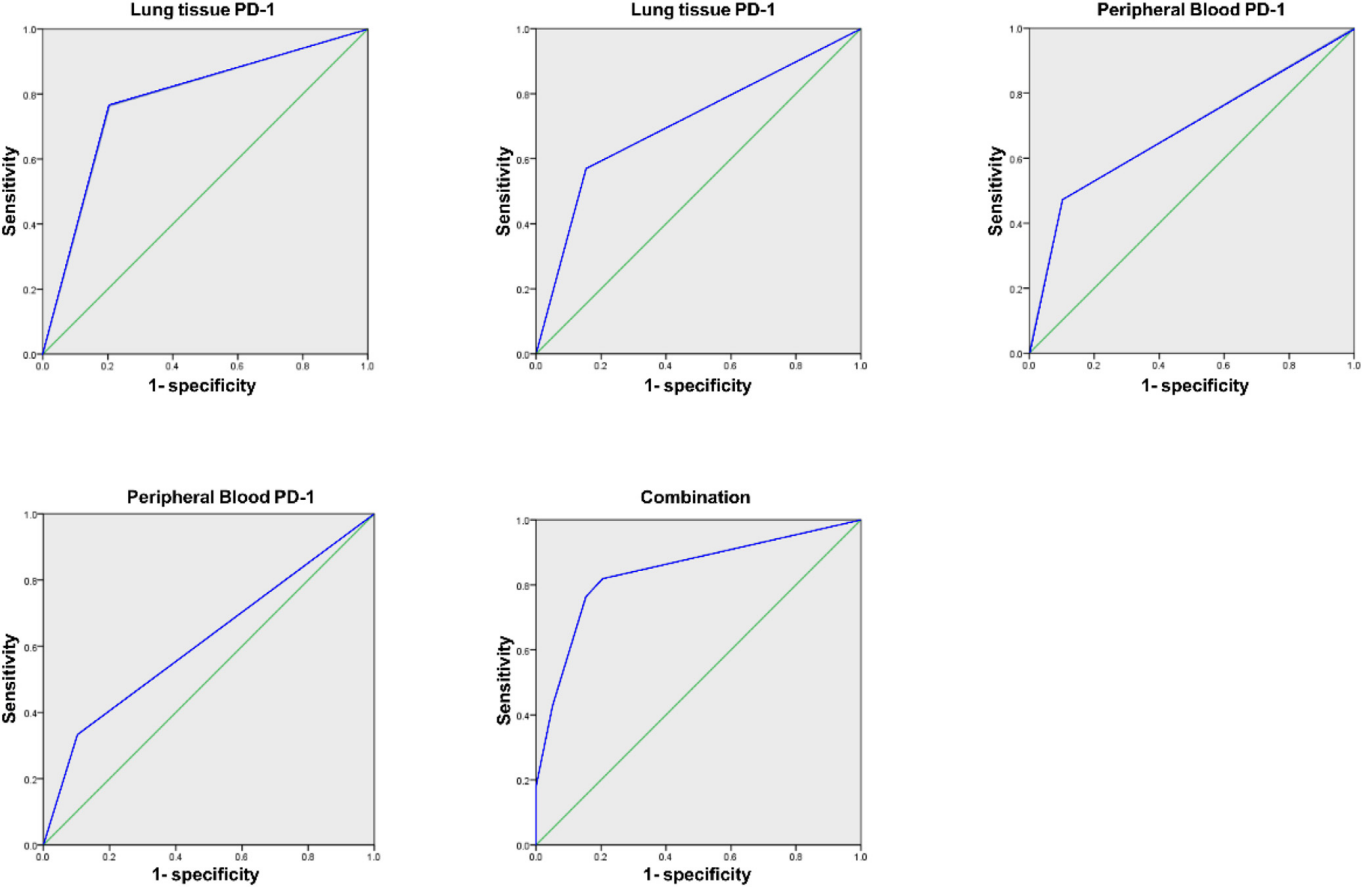
PD-1/PDL-1′S ROC curve analysis in lung tissue and PPB in LC's diagnosis.

Area Under the Curve (AUC) of PD-1 and PDL-1 in lung tissue and PPB of LC of combined diagnosis was elevated vs. each index's alone examination (*p* < 0.05), as manifested in Table 3 and Fig. 1.

**Table 3:** Analysis of PD-1/PDL-1′s diagnostic value in lung tissue and PPB for LC.

Index	AUC	SE	95 % CI
Lung tissue PD-1	0.779	0.047	0.686∼0.872
Lung tissue PDL-1	0.708^a^	0.050	0.609∼0.806
PPB PD-1	0.685^a^	0.051	0.586∼0.784
PPB PDL-1	0.615^a^	0.054	0.510∼0.721
Combination	0.841	0.039	0.765∼0.917

Vs. the combination.

a *p <* 0.05.

### *PD-1/PDL-1′s comparison in cancer tissues and PPB of the remission with the non-remission*

PD-1 and PDL-1′s positive rate in cancer tissues and PPB in remission was declined vs. the non-remission (*p* < 0.05), as presented in Table 4.

**Table 4:** Comparison of PD-1/PDL-1 in cancer tissues and PPB of the remission and the non-remission (cases, %).

Groups	n	Cancer tissue PD-1	Cancer tissue PDL-1	PPB PD-1	PPB PDL-1
The non-remission	27	26 (96.30)	24 (88.89)	19 (70.37)	13 (48.15)
The remission	45	29 (64.44)	17 (37.78)	15 (33.33)	11 (24.44)
*χ^2^*		9.492	17.980	9.288	4.267
P		0.002	< 0.001	0.002	0.039

### *Analysis of PD-1/PDL-1′s predictive value in cancer tissue and PPB on immunotherapy's efficacy*

**Fig. 2:**
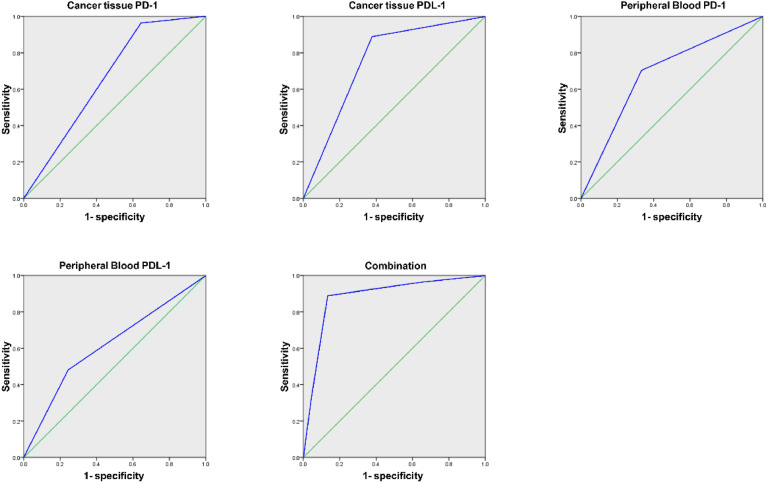
PD-1/PDL-1′S ROC curve analysis in cancer tissues and PPB to predict immunotherapy's efficacy.

AUC of the joint test in assessing immunotherapy's efficacy was augmented vs. each index's alone examination (*p* < 0.05), as presented in Table 5 and Fig. 2.

**Table 5:** Analysis of PD-1/PDL-1′s predictive value in cancer tissues and PPB on immunotherapy's efficacy.

Index	AUC	SE	95 % CI
Cancer tissue PD-1	0.659^a^	0.063	0.535∼0.784
Cancer tissue PDL-1	0.756^a^	0.058	0.642∼0.869
PPB PD-1	0.685^a^	0.065	0.557∼0.814
PPB PDL-1	0.619^a^	0.07	0.482∼0.756
Combination	0.884	0.044	0.798∼0.971

Vs. the combination.

a *p <* 0.05.

### *PD-1/PDL-1 in cancer tissues and PPB in the death and the survival*

PD-1 and PDL-1′s positive rate in cancer tissues and PPB in the death was elevated vs. the survival (*p* < 0.05), as manifested in Table 6.

**Table 6:** PD-1/PDL-1 in cancer tissues and PPB of the death and the survival.

Groups	n	Cancer tissue PD-1	Cancer tissue PDL-1	PPB PD-1	PPB PDL-1
The death	33	32 (96.97)	31 (93.94)	21 (63.64)	17 (51.52)
The survival	39	23 (58.97)	11 (28.21)	13 (33.33)	7 (17.95)
*χ^2^*		14.307	31.778	6.586	9.063
p		< 0.001	< 0.001	0.010	0.003

### *Analysis of PD-1/PDL-1′s predictive value in cancer tissue and PPB for patients’ prognosis*

**Fig. 3:**
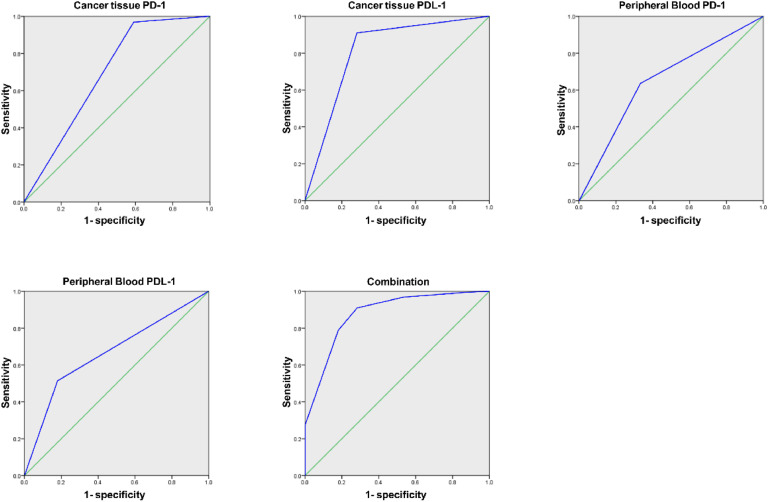
PD-1/PDL-1′S ROC curve analysis in cancer tissues and PPB to predict patients’ prognostic.

AUC of joint examination in predicting LC patients’ prognosis was elevated vs. each index's alone detection (*p* < 0.05), as manifested in Table 7 and Fig. 3.

**Table 7:** Analysis of PD-1/PDL-1′s predictive value in cancer tissues and PPB on patients’ prognosis.

Index	AUC^a^	SE	95 % CI
Cancer tissue PD-1	0.690	0.062	0.568∼0.812
Cancer tissue PDL-1	0.814	0.053	0.710∼0.917
PPB PD-1	0.652^a^	0.066	0.523∼0.780
PPB PDL-1	0.668^a^	0.065	0.540∼0.796
Combination	0.878	0.040	0.799∼0.957

Vs. the combination.

a *p <* 0.05.

### *Comparison of patients’ 3-year survival rate with PD-1/PDL-1 in diverse cancer tissues*

**Fig. 4:**
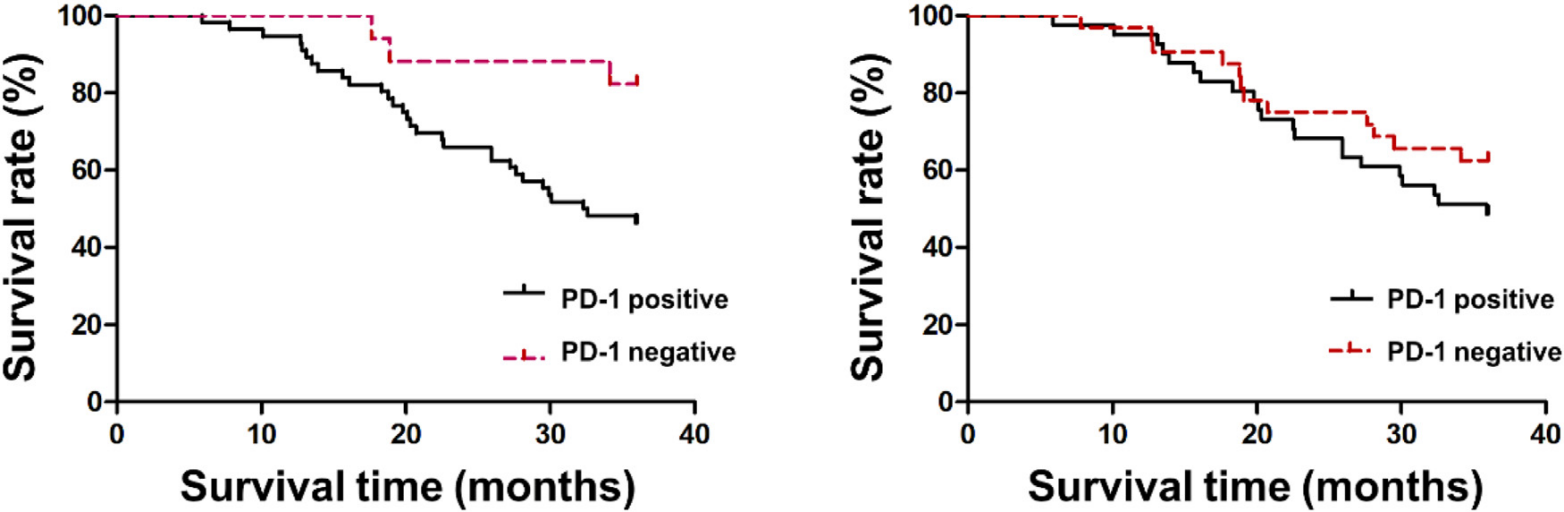
Comparison of patients’ 3-year survival rates with PD-1/PDL-1 in diverse cancer tissues.

Patients’ 3-year survival rate with PD-1 positive was 45.45 % (25/55), which was declined vs. the negative patients 82.35 % (14/17) (*p* < 0.05). PDL-1′s 3-year survival rates in cancer tissue in the positive and negative were 48.78 % (20/41) and 61.29 % (19/31), separately, and no distinct differences were presented between the two (*p* > 0.05), as manifested in Fig. 4.

## Discussion

LC is tumor-linked death's primary reason all over the world. Patients’ asymptomatic period lasts for a long time owing to its insidious onset and easy confusion with benign pulmonary illnesses; Multiple patients have been diagnosed with distant metastasis, losing surgical cure opportunities, and thus influencing their prognosis.^9^^,^^10^ Relevant reports have elaborated that LC's occurrence and advancement are linked with T cell immune deficiency in the body.^11^ PD-1/PDL-1, a co-stimulus analysis with negative regulatory function in the B7 family, implicates in tumor immune escape mechanism via negative signal's limitation, T-cell response's termination and attenuation, thereby boosting tumor cell growth.^12^^,^^13^ Interrelated reports have illuminated PD-1/PDL-1 in LC tissues is distinctly elevated.^14^ In this study, PD-1 and PDL-1′s positive rates in lung tissue and PPB in the LC were augmented vs. the benign, clarifying that PD-1 and PDL-1′s positive rates in lung tissue and PPB in LC patients were elevated, which was basically associated with the fact that PD-1/PDL-1 was available to implicate in LC patients’ immune modulation and the interaction between the two was available to mediate immune escape control in the engine body.^15^ This research's results elucidated AUC of PD-1 and PDL-1 in lung tissue and PPB of LC's joint diagnosis was elevated vs. alone examination of each index, clarifying that combined test was provided with diagnostic value for LC.

Presently, LC's therapy strategies primarily cover radiotherapy, chemotherapy, targeted therapy and immunotherapy, etc. Meanwhile, as tumor immunology advances, immunotherapy has been paid extensive attention in diversified tumor illnesses.^16^ Immunotherapy for lung cancer is a therapeutic method to help the immune system recognize and clear cancer cells.^17^ Associated reports have illuminated the balance of tumor cells with the immune system exerts crucial action in tumors’ occurrence and progression.^18^ The nature of the interaction between tumor cells and immune cells in the tumor microenvironment determines the antitumor response. Recent studies have shown that immune cells play a dichotomous role in the evolution of lung tumors, which can not only promote anti-tumor responses but also regulate the tumor microenvironment of immunosuppression (pro tumorigenesis).^19^ Immune escape, the prerequisite for tumor cells’ generation, overcomes the immune state, activates the patient's own immune system and strengthens cellular immunity and humoral immune response in the engine body via introducing tumor antigens into patients in multiple forms, thus exerting controlling and eliminating tumor cells’ actions.^20^^,^^21^ Nevertheless, numerous researches have elucidated that immunotherapy's effect varies greatly among diverse patients, and several reports have manifested that immunotherapy's effect is linked with PD-1 in cancer tissues.^22^^,^^23^ The PCD-1/PD-Ls pathway plays a fundamental role in manipulating the magnitude of T-cell responses, regulating their activation, and generating immune tolerance in the Tumor Microenvironment (TME) and peripheral tissues.^8^ Previous studies showed that anti-PD-L1 antibody treatment effectively inhibited the growth of pancreatic tumors by activating cytotoxic T-cells, and its anti-tumor immune mechanism mainly depended on the PD-L1 pathway and CD8 + T*-*cells.^24^ Consequently, relevant research in this study clarified PD-1 and PDL-1′s positive rates in cancer tissues and PPB in remission were declined vs. the non-remission, elaborating that immunotherapy’ efficacy was associated with it, which might be linked with the enhancement of tumor cells’ immune escape via PD-1 and PDL-1′s positive.

Additionally, the results of this research elaborated AUC of the joint test in assessing immunotherapy's efficacy was elevated vs. each index's alone examination, clarifying that the combined test was provided with appraisal value for immunotherapy's efficacy.

LC's good prognosis primarily relies on early diagnosis and cure, while immunotherapy's application is available to distinctively prolong patients’ survival and enhance patients’ quality of life.^25^ Additionally, this research's above results illuminated that PD-1 and PDL-1 were nearly associated with immunotherapy's efficacy, elaborating that PD-1 and PDL-1 in patients might be associated with prognosis. The results elucidated PD-1 and PDL-1′s positive rates in cancer tissues and PPB in the death were elevated vs. the survival, clarifying that patients with PD-1 and PDL-1′s positive in cancer tissues and PPB were provided with unpleasing prognosis. The reason is that PD-1 is an inhibitory costimulatory molecule and PDL-1 is a stromal cell in tumor cells and microenvironment. The combination of the two is available to restrain T-cells’ function to lead to immune escape's occurrence, thereby accelerating tumor cell growth.^26^ Additionally, this study elaborated that patients’ 3-year survival rate with positive PD-1 in cancer tissues declined vs. the negative patients, which further testified PD-1 in cancer tissues was linked with patients’ prognosis. Relevant research has illuminated PD-1 in LC tissues is available to influence T-cell activation to decline cytotoxic T-cells’ tumor-killing efficacy, thus influencing patients’ prognosis.^27^ Moreover, PD-L1 expression in tumors or infiltrating immune cells has been demonstrated in a variety of tumors by immunohistochemistry, indicating the role of the PD-1/PD-L1 axis as a prognostic feature and therapeutic target in multiple tissue types. Combining previous studies with these results, PD-L1 can serve as a predictive biomarker for the response to PD-1/PD-L1 inhibitors.^8^ While this study's results elaborated PD-1 was not associated with prognosis in cancer tissues in LC patients, which was not assisted with the above research results, which might be linked with this research's few sample sizes. Consequently, the sample size should be elevated later to analyze PD-1 and prognosis in the cancer tissues in LC patients. Furthermore, it is of high clinical value to further explore the quality of life of LC patients after immunotherapy.

In brief, aberrant PD-1 with PDL-1 in cancer tissues and PPB was presented in LC patients, and PD-1 with PDL-1′s combined test in cancer tissues and PPB was provided with diagnostic value for LC and assessment value for immunotherapy's efficacy with prognosis.

## Availability of data and materials

The datasets used and/or analyzed during the present study are available from the corresponding author on reasonable request.

## Ethical approval

All procedures performed in this study involving human participants were in accordance with the ethical standards of the institutional and/or national research committee and with the 1964 Helsinki Declaration and its later amendments or comparable ethical standards. All subjects were approved by Shanxi Provincial People's Hospital (Approval nº 201311S51).

## Authors’ contributions

Jun Ma designed the research study. JianRui Song and Lina Han performed the research. Wen Zhou, LiFeng Meng and JianHui Li provided help and advice. JianRui Song, Wen Zhou and XiaoMing Bai analyzed the data. Jun Ma wrote the manuscript. Jun Ma reviewed and edited the manuscript. All authors contributed to editorial changes in the manuscript. All authors read and approved the final manuscript.

## Funding

Shanxi Provincial Basic Research Program (Free Exploration): Natural Science Research (202103021224383).

## Declaration of competing interest

The authors declare no conflicts of interest.

## References

[1] Cho Y., Park S., Byun H.K., Lee C.G., Cho J., Hong M.H. (2019). Impact of treatment-related lymphopenia on immunotherapy for advanced non-small cell lung cancer. Int J Radiat Oncol Biol Phys.

[2] Hamilton G., Rath B. (2019). Immunotherapy for small cell lung cancer: mechanisms of resistance. Expert Opin Biol Ther.

[3] Yvorel V., Patoir A., Casteillo F., Tissot C., Fournel P., Stachowicz M.-L. (2017). PD-L1 expression in pleomorphic, spindle cell and giant cell carcinoma of the lung is related to TTF-1, p40 expression and might indicate a worse prognosis. PLoS One.

[4] Yokobori T., Yazawa S., Asao T., Nakazawa N., Mogi A., Sano R. (2019). Fucosylated α1-acid glycoprotein as a biomarker to predict prognosis following tumor immunotherapy of patients with lung cancer. Sci Rep.

[5] Guillon A., Reckamp K.L. (2017). Heuzé-Vourc'h N. Immunotherapy improves the prognosis of lung cancer: do we have to change intensive care unit admission and triage guidelines?. Crit Care.

[6] Guo L., Song P., Xue X., Guo C., Han L., Fang Q. (2019). Variation of programmed death ligand 1 expression after platinum-based neoadjuvant chemotherapy in lung cancer. J Immunother.

[7] Chen W.-X., Li G.-X., Hu Z.-N., Zhu P., Zhang B.-X., Ding Z.-Y. (2019). Significant response to anti-PD-1 based immunotherapy plus lenvatinib for recurrent intrahepatic cholangiocarcinoma with bone metastasis: a case report and literature review. Medicine (Baltimore).

[8] Gutic B., Bozanovic T., Mandic A., Dugalic S., Todorovic J., Stanisavljevic D. (2023). Programmed cell death-1 and its ligands: current knowledge and possibilities in immunotherapy. Clinics (Sao Paulo).

[9] Leng C., Li Y., Qin J., Ma J., Liu X., Cui Y. (2016). Relationship between expression of PD-L1 and PD-L2 on esophageal squamous cell carcinoma and the antitumor effects of CD8+ T cells. Oncol Rep.

[10] Yildiz I., Bahsi S., Erdamar S., Goksel S., Demir G., Er O. (2019). Clinicopathological and prognostic significance of microsatellit instability (MSI) status and PDl-1 expression in Turkish patients with gastric cancer. J Clin Oncol.

[11] Yinxing Fan, Rong Chai, Jiayi Zhao (2019). Expression of Ki-67 and PD-L1 in non-small cell lung cancer and its effect on prognosis. Chinese J Cancer Biotherap.

[12] Berntsson J., Eberhard J., Nodin B., Leandersson K., Larsson A.H., Jirström K. (2018). Expression of programmed cell death protein 1 (PD-1) and its ligand PD-L1 in colorectal cancer: relationship with sidedness and prognosis. Oncoimmunology.

[13] Jiang Bo, Zhu Ying, Tu Changling (2018). Effect of gemcitabine on RRM1 expression in peripheral blood of elderly patients with advanced non-small cell lung cancer. Chinese J Modern Med.

[14] Zhang M.L., Kem M., Mooradian M.J., Eliane J.-P., Huynh T.G., Iafrate A.J. (2019). Differential expression of PD-L1 and IDO1 in association with the immune microenvironment in resected lung adenocarcinomas. Mod Pathol.

[15] Chang H., Hong J.H., Lee J.K., Cho H.W., Ouh Y.T., Min K.J. (2018). Programmed death-1 (PD-1) expression in cervical intraepithelial neoplasia and its relationship with recurrence after conization. J Gynecol Oncol.

[16] Xing Y.F., Pan X., Qian B., Shi M.H. (2019). Expression of PD-1 and PD-L1 in peripheral blood of patients with advanced non-small cell lung cancer. Zhonghua Yi Xue Za Zhi.

[17] Lahiri A., Maji A., Potdar P.D., Singh N., Parikh P., Bisht B. (2023). Lung cancer immunotherapy: progress, pitfalls, and promises. Mol Cancer.

[18] Zhao Ning, Ren Hongliang, Pan Na (2017). Expression of PD-L1 in non-small cell lung cancer and its relationship with clinical factors. Chin J Cancer Biotherap.

[19] Friedlaender A., Banna G.L., Buffoni L., Addeo A. (2019). Poor-performance status assessment of patients with non-small cell lung cancer remains vague and blurred in the immunotherapy era. Curr Oncol Rep.

[20] Genova C., Dellepiane C., Carrega P., Sommariva S., Ferlazzo G., Pronzato P. (2022). Therapeutic implications of tumor microenvironment in lung cancer: focus on immune checkpoint blockade. Front Immunol.

[21] Wang Lingling, Gao Ying, Shen Bing (2017). Expression of PD-L1 in lung squamous cell carcinoma and its relationship with clinicopathological parameters and prognosis. Shandong Med J.

[22] Takamori S., Toyokawa G., Takada K., Shoji F., Okamoto T., Maehara Y. (2018). Combination therapy of radiotherapy and anti-PD-1/PD-L1 treatment in non-small-cell lung cancer: a mini-review. Clin Lung Cancer.

[23] Kerr K.M., Thunnissen E., Dafni U., Finn S.P., Bubendorf L., Soltermann A. (2019). A retrospective cohort study of PD-L1 prevalence, molecular associations and clinical outcomes in patients with NSCLC: results from the European Thoracic Oncology Platform (ETOP) Lungscape Project. Lung Cancer.

[24] Yang H., Zhang X., Lao M., Sun K., He L., Xu J. (2023). Targeting ubiquitin-specific protease 8 sensitizes anti-programmed death-ligand 1 immunotherapy of pancreatic cancer. Cell Death Differ.

[25] Dall'Olio F.G., Gelsomino F., Conci N., Marcolin L., De Giglio A., Grilli G. (2021). PD-L1 expression in circulating tumor cells as a promising prognostic biomarker in advanced non-small-cell lung cancer treated with immune checkpoint inhibitors. Clin Lung Cancer.

[26] Young S., Griego-Fullbright C., Wagner A., Chargin A., Patterson B.K., Chabot-Richards D. (2018). Concordance of PD-L1 expression detection in non-small cell lung cancer (NSCLC) tissue biopsy specimens between oncotect iO lung assay and immunohistochemistry (IHC). Am J Clin Pathol.

[27] Fumet J.D., Richard C., Ledys F., Klopfenstein Q., Joubert P., Routy B. (2019). Correction: prognostic and predictive role of CD8 and PD-L1 determination in lung tumor tissue of patients under anti-PD-1 therapy. Br J Cancer.

